# A Sensitive Ratiometric Fluorescent Sensor for Zinc(II) with High Selectivity

**DOI:** 10.3390/s130303131

**Published:** 2013-03-06

**Authors:** Yuanyuan Lv, Mingda Cao, Jiakai Li, Junbo Wang

**Affiliations:** School of Medicine, Zhejiang University City College, Hangzhou 310015, Zhejiang, China; E-Mails: cmd92427@163.com (M.C.); 13819162200@163.com (J.L.)

**Keywords:** fluorescent sensor, ratiometric sensor, porphyrin, zinc ion

## Abstract

A new fluorescent Zn^2+^ chemosensor (P1) based on a functionalized porphyrin was synthesized and characterized. P1 displayed dramatic ratiometric variations in absorption and fluorescent emission spectra upon exposure to Zn^2+^ due to the formation of a 1:1 Zn^2+^/P1 complex. The sensor also exhibited high selectivity and sensitivity toward Zn^2+^ over other common metal ions in the physiological pH range with a detection limit of 1.8 μM. The sensor showed fast response times and excellent reproducibility, thus confirming its potential applicability as a fluorescent sensor for Zn^2+^ sensing.

## 1. Introduction

Zinc ions (Zn^2+^), the second most abundant transition-metal ions in the human body, play diverse roles in biological processes, such as gene expression, metalloenzyme function, and neurotransmission [1–3]. Though zinc is a relatively nontoxic element, it can be toxic if consumed in large enough quantities. For example, zinc is a metal pollutant of environment, significant concentrations of which may reduce the soil microbial activity causing phytotoxic effects and it is a common contaminant in agricultural and food wastes. Consequently, the exploitation of chemosensors with high selectivity and sensitivity for detecting trace amounts of Zn^2+^ has attracted increased research interest. Given that Zn^2+^ does not produce spectroscopic or magnetic signals because of its 3d^10^4s^0^ electronic configuration, the fluorescence method is the primary method of choice for Zn^2+^ determination [4,5]. Several studies have reported the successful application of fluorescent sensors based on quinoline [6,7], fluorescein [8,9], coumarin [10], indole [11], naphthalimide [12], and peptides [13] in the detection of Zn^2+^. However, the development of new small molecular fluorescent sensors with extremely high sensitivity for Zn^2+^ and high selectivity over other relevant metal ions remains desirable.

**Scheme 1.**
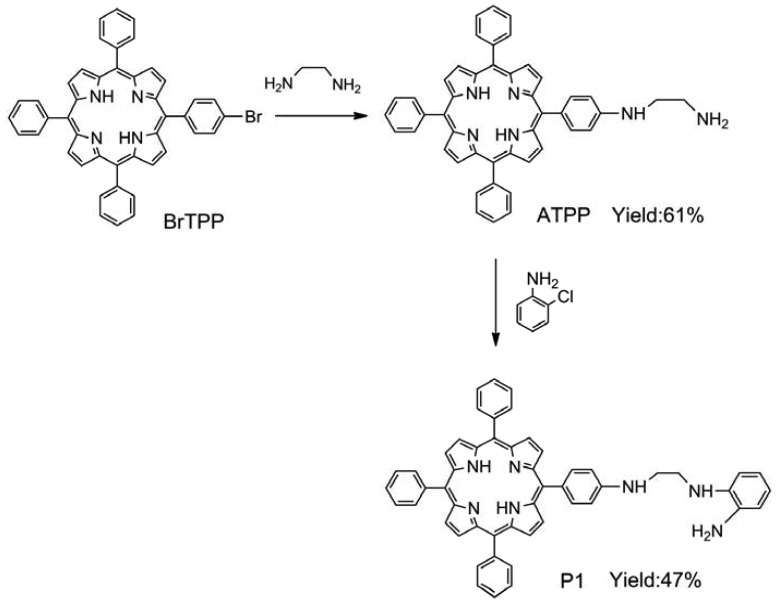
Schematic of P1 synthesis.

Among various fluorescent sensors currently available, porphyrin and its derivatives are the first class of probes to be developed for Zn^2+^. These organic compounds exhibit good photostability, high absorption coefficients in the region from 400 nm to 450 nm (visible range), large Stokes shifts that minimize the effects of background fluorescence, and appreciable changes in spectral shift upon ligand binding [14–17]. Ratiometric fluorescence measurements have recently been conducted to detect metal ions. The method involves the observation of changes in the ratio of emissions at two wavelengths, thereby increasing the selectivity and sensitivity of determination and eliminating most or all of the possible variabilities arising from differences in instrumental efficiency and environmental effects [18–24]. In this study, we report a novel ratiometric fluorescent probe P1 for Zn^2+^ based on a porphyrin derivative (see Scheme 1). To achieve the requirement of high selectivity toward Zn^2+^, a strong chelator bearing three nitrogen atoms was incorporated into the porphyrin. These nitrogen atoms can form a cavity that can suitably contain Zn^2+^. The amine group of the chelator is a hydrophilic group, which can enhance the water solubility of the sensor [25].

## 2. Experimental Section

### 2.1. Materials and Apparatus

All chemicals were purchased from commercial suppliers. Methanol, ethanol, and acetonitrile were dried and distilled before use. *N*,*N*′-dimethylformamide (DMF) of analytical reagent grade was dried over 4 Å molecular sieves. All other chemicals were of analytical reagent grade and used without further purification, unless otherwise specified. 5-(4-Bromophenyl)-10,15,20-triphenylporphyrin (BrTPP) was synthesized using a reported procedure [26]. The ^1^H-NMR spectra were obtained on a Bruker (Advance DMX500) NMR spectrometer. Mass spectra (MS) were recorded with a Bruker Esquire 3000 Plus spectrophotometer (Bruker-Franzen Analytik GmbH, Bremen, Germany). IR spectra were recorded on a spectrometer (Nicolet, Nexus-470, Madison, WI, USA) for the compounds in the solid state, prepared as KBr discs. Ultraviolet visible (UV−vis) absorption spectra were obtained using a Shimadzu UV 2450 spectrophotometer (Shimadzu, Kyoto, Japan). Fluorescence spectra measurements were performed on a Shimadzu RF-5301 PC (Shimadzu) fluorescence spectrophotometer. Both excitation and emission slits measured 3 nm. All pH measurements were made with a pH-10C digital pH meter. All titration experiments were run twice to generate reliable data.

### 2.2. Synthesis of P1

BrTPP (2.08 g, 3 mmol) and ethylenediamine (6.0 mL, 90 mmol) were dissolved in DMF (15 mL). Then CuSO_4_·5H_2_O (0.1 g, 0.4 mmol) was added to this solution. The resulting mixture was heated under reflux for 5 h. After cooling to room temperature, the solution was poured into water (100 mL). The precipitate was collected by filtration, washed with water, and dried to yield a purple powder. The product was purified by silica gel column chromatography using dichloromethane/methanol (15:1, v/v) as an eluent to afford 5-[4-(aminoethylene)amino]-10,15,20-triphenylporphyrin (ATPP). Yield: 61%. ^1^H-NMR (CDCl_3_, δ): 8.81 (s, 4H), 8.65 (s, 4H), 8.29 (m, 3H), 7.67–7.80 (m, 12H), 7.61–7.67 (m, 4H), 6.98 (m, 1H), 4.12 (s, 2H), 3.59 (m, 2H), 2.68 (m, 2H), −2.76 (s, 2H); MS (ESI): 673.2 [M + 1] ^+^; IR (KBr, ν) 3,306, 3,267, 3,227, 3,005, 2,926, 1,080, 899 cm^−1^ (see Figures S1–S3 in Supplementary Material). For P1 synthesis, ATPP (0.404 g, 0.6 mmol), *ortho*-aminophenol (0.055 g, 0.5 mmol), and potassium iodide (0.008 g, 0.05 mmol) were added to an acetonitrile solution (30 mL). The reaction mixture was stirred and heated by refluxing for 6 h under a nitrogen atmosphere. After removal of the solvent, the crude mixture was purified by silica gel column chromatography using dichloromethane/methanol (12:1, v/v) as the eluent, thus yielding compound P1. Yield: 47%. ^1^H-NMR (CDCl_3_, δ): 8.83 (s, 4H), 8.70 (s, 4H), 8.31 (m, 3H), 7.61–7.71 (m, 12H), 7.57–7.59 (m, 4H), 7.06 (s, 2H), 6.74–6.92 (m, 4H), 5.32 (s, 2H), 3.94 (m, 4H), −2.81 (s, 2H); MS (ESI): 764.3 [M + 1] ^+^; IR (KBr, ν) 3,452, 3,417, 3,356, 3,030, 2,912, 1,489, 867 cm^−1^ (see Figures S4–S6 in Supplementary Material).

### 2.3. Measurement Procedures

Stock solutions of 20 μM P1 and 1.0 × 10^4^ μM Zn^2+^ were prepared by dissolving P1 in absolute ethanol and ZnSO_4_·7H_2_O in doubly distilled water, respectively. The latter solution was further diluted stepwise to yield working solutions with concentrations ranging from 1.0 × 10^3^ μM to 1.0 μM. The wide-pH range solutions were prepared by adjusting 5.0 × 10^4^ μM Tris–HCl solution with HCl or NaOH solution. Solutions of other metal ions were prepared by dissolving NaCl, KCl, CaCl_2_, Cd(NO_3_)_2_·4H_2_O, Cr(NO_3_)_3_·9H_2_O, Co(NO_3_)_2_·6H_2_O, MgSO_4_, Cu(NO_3_)_2_·3H_2_O, Pb(NO_3_)_2_, MnSO_4_·H_2_O, FeSO_4_·7H_2_O and HgCl_2_ in doubly distilled water (1.0 × 10^3^ μM).

A complex solution of Zn^2+^/P1 was prepared by adding 5.0 mL of the stock solution of P1 and 1.0 mL of the stock solution of Zn^2+^ to a 10.0 mL volumetric flask. The mixture was then diluted to 10.0 mL with Tris–HCl buffer solution (pH 7.4). The final concentrations of P1 and Zn^2+^ in the complex solutions were 10 μM and 100 μM–0.1 μM, respectively. The solution was protected from light and kept at 4 °C until use. A blank solution of P1 was prepared under the same conditions without Zn^2+^.

## 3. Results and Discussion

### 3.1. Preparation and Characterization of P1

**Figure 1.**
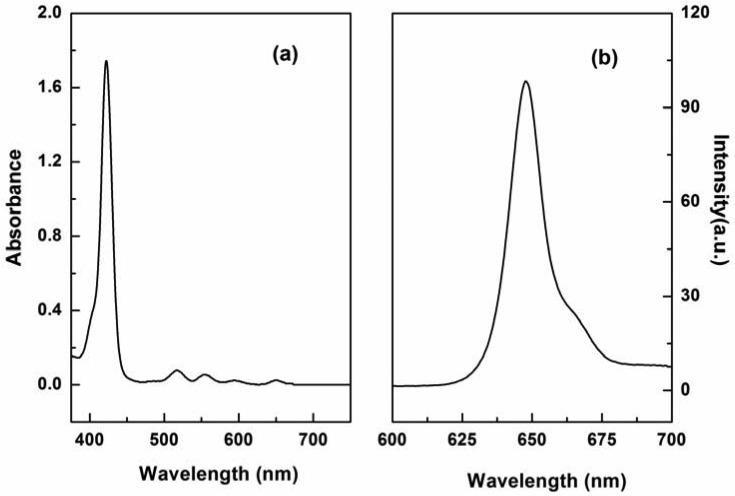
Absorption (**a**) and (**b**) fluorescence emission spectra of the P1 solution (10 μM).

P1 was easily synthesized with 47% yield by a two-step procedure [27,28]. The absorption spectrum of P1 displayed the characteristic transitions of porphyrin with an intense Soret band at 422 nm and four weak Q-bands at 519, 555, 594, and 650 nm (see Figure 1(a)). This result is in agreement with our finding that P1 exhibits a typical fluorescence peak at 650 nm when excited at 420 nm (see Figure 1(b)). Tetraphenylporphyrin was used as a standard (Φ*_ref_* = 0.15) [29], and the fluorescence quantum yield (Φ*_F_*) of P1 in *N*,*N*′-dimethylacetamide was determined to be 0.20, according to the method described by Demas and Crosby [30].

### 3.2. Sensing Properties of P1 to Zn^2+^

**Figure 2.**
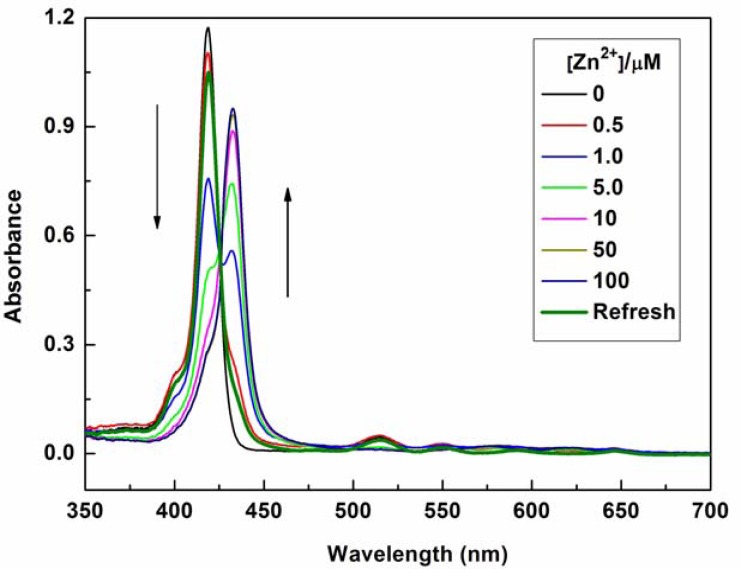
UV–vis spectra of P1 (10 μM) at pH 7.4 in the presence of different concentrations of Zn^2+^ (0, 0.5, 1.0, 5.0, 10, 50, and 100 μM) in EtOH/H_2_O solution (1:1, v/v) or recovered with addition of EDTA.

**Figure 3.**
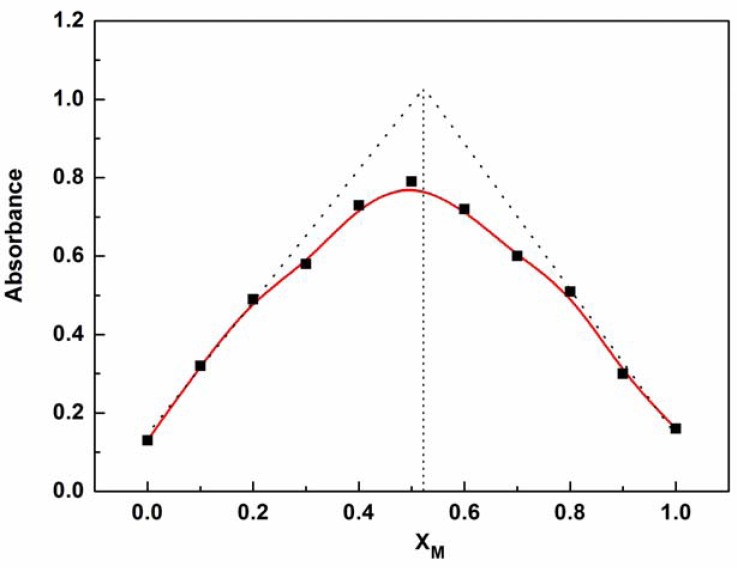
Job's plot for the determination of the binding stoichiometry of P1 with Zn^2+^ obtained from variations in absorption at 436 nm.

The mode of coordination of P1 with Zn^2+^ was investigated by spectrophotometric titration. Upon the gradual addition of Zn^2+^ (0.0–10.0 equiv.) to P1 (10 μM) solution, the Soret band of P1 showed a gradual bathochromic shift from 422 nm to 436 nm with an isosbestic point at 426 nm and a decrease in the Q*_y_*(0–0) band at 519 nm (see Figure 2). This result indicates that a new species is only produced during the titration [21–24,31–36]. The changes in the UV–vis spectrum of the solutions can be interpreted as the electron rearrangement in P1 through strong Zn^2+^ coordination. To further understand the binding behavior and determine the stoichiometry of the formed complex, the Job's plot for the absorbance was determined by keeping the sum of initial concentrations of Zn^2+^ and P1 constant at 10 μM and changing the molar ratio of Zn^2+^ (X_M_ = ([Zn^2+^]/([Zn^2+^] + [P1])) from 0 to 1. As shown in Figure 3, a plot of absorbance at 436 nm *versus* X_M_ shows that the absorbance value is highest at a molar fraction of ca. 0.5, indicating that the complex formed between P1 and Zn^2+^ follows a 1:1 stoichiometry [37,38].

**Figure 4.**
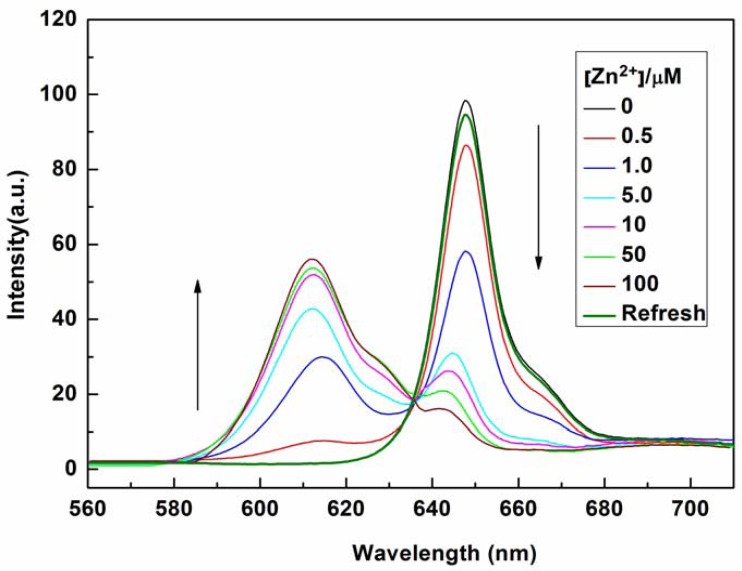
Fluorescence emission spectra of P1 (10 μM) at pH 7.4 in the presence of different concentrations of Zn^2+^ (0, 0.5, 1.0, 5.0, 10, 50, and 100 μM) in EtOH/H_2_O (1:1, v/v) solution or recovered with addition of EDTA.

**Scheme 2.**
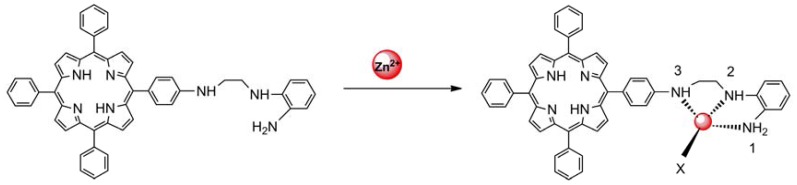
Proposed binding mode of P1 with Zn^2+^.

The sensing properties of P1 towards Zn^2+^ were also investigated by emission spectral titration. Figure 4 reveals that P1 could be developed as a ratiometric sensor for Zn^2+^. Upon the gradual addition of Zn^2+^ (0.0–10.0 equiv.) to a P1 solution (10 μM), the emission peak of P1 at 650 nm decreased with the concomitant formation of new peak at 610 nm. When Zn^2+^ ions were further added to the P1 solution, the ratiometric change in fluorescence spectra became evident with a clear isoemission point at 636 nm. The Zn^2+^-induced emission shift of P1 also confirmed the formation of a Zn^2+^/P1 complex. To investigate the binding behavior of P1 with Zn^2+^, ^1^H-NMR spectra of P1 in the absence and presence of Zn^2+^ were recorded at room temperature as shown in Figure S7. Upon addition of 1.0 equiv of Zn^2+^, the protons signals for the H2, H3 and NH_2_ groups underwent distinct downfield shifts due to the deshielding effect of the metal ion, implying that the three N atoms (labeled N1, N2, and N3 in Scheme 2) should be involved directly in Zn^2+^ coordination. In addition, the coordination of Zn^2+^ by the N2, N3 atoms promoted a downfield shift for all aromatic protons (H4-H7) in the phenyl ring of the ligating group. Thus, we can deduce a possible coordination mode between Zn^2+^ and P1, as shown in Scheme 2. The three N atoms (labeled N1, N2, and N3 in Scheme 2) participate in the coordination with Zn^2+^. The fourth ligand (X) was considered to be SO_4_^2−^ by consideration of the charge balance in the solution [3,39]. Plotting the concentrations of Zn^2+^ against the variation in absorbance (*ΔA*), which refers to the difference in the absorbance intensities of P1 at 436 nm before and after exposure to various concentrations of Zn^2+^, yields the following equation [40]:

(1) Absorbance=0.113×concentration+0.152

(correlation coefficient = 0.9963). The calculated detection limit was 1.8 μM.

We investigated the time evolution of the response of P1 to 2.0 equiv. of Zn^2+^ in EtOH/H_2_O solution (1:1, v/v) (see Figure S8 in Supplementary Material). The interaction of P1 with Zn^2+^ was completed in less than 2 min. Therefore, this system could be used for real-time tracking of Zn^2+^.

The optical responses of P1 to Zn^2+^ were found to be fully reversible. When ethylene diamine tetraacetic acid (EDTA) was subsequently added to the complexed solution of P1 and Zn^2+^, an absorption peak at 422 nm and a fluorescence signal at 650 nm were completely recovered. This result demonstrates that the binding between P1 and Zn^2+^ is chemically reversible and not a cation-catalyzed reaction (see Figures 2 and 4).

### 3.3. Effect of pH

**Figure 5.**
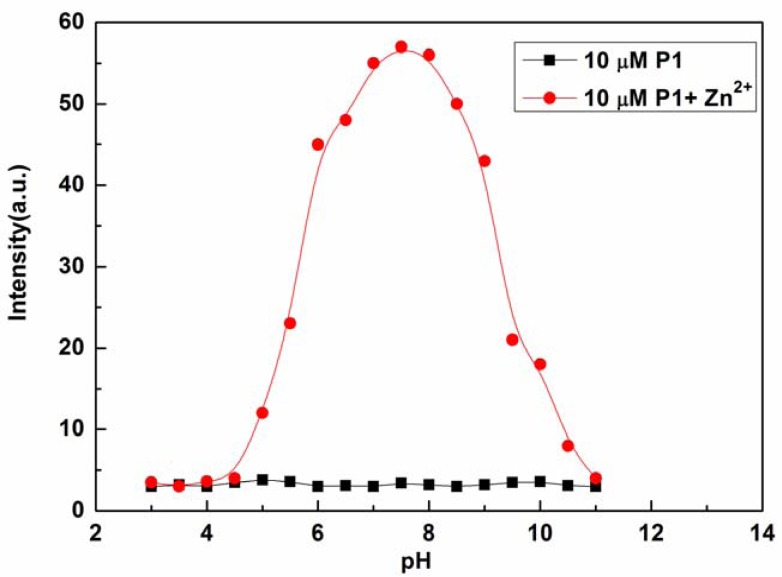
Fluorescence intensities of P1 (10 μM) at 610 nm in the absence and presence of Zn^2+^ (10 μM) at various pH values, *λ*_ex_ = 420 nm.

The effects of pH on the fluorescence intensity of P1 at 610 nm in the presence of Zn^2+^ were determined in the pH range from 3.0 to 11.0 with the concentration of Zn^2+^ fixed at 10 μM (see Figure 5). The fluorescence intensity of P1 at 610 nm decreased with decreasing pH value, which may be caused by the protonation of P1. On the other hand, high pH values led to the precipitation of Zn(OH)_2_, which, in turn, reduced complexation with P1. At pH values ranging from 6.5 to 8.5, acidity did not appear to affect the determination of Zn^2+^ with P1 [41]. Therefore, the response behavior of P1 is pH independent within the relevant biological pH range. These results indicate that the proposed sensor is convenient for practical applications of Zn^2+^ determination from actual samples.

### 3.4. Selectivity

**Figure 6.**
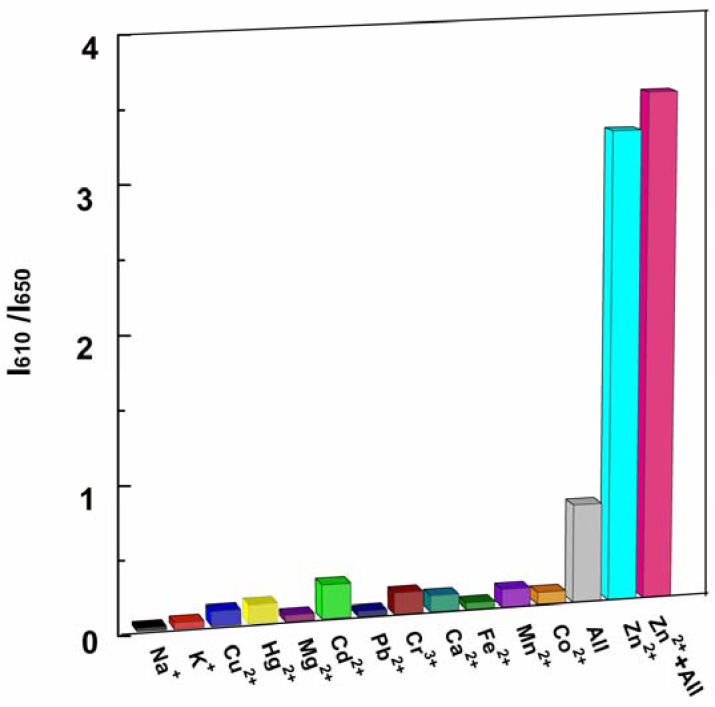
Changes in the fluorescence ratio (I_610 nm_/I_650 nm_) of P1 (10 μM) at pH 7.4 to Zn^2+^ (10 μM) and other metal ions (each 1.0 × 10^3^ μM), *λ*_ex_ = 420 nm.

High selectivity is a necessary feature of excellent chemosensors. The selectivity of P1 to Zn^2+^ ions and competition with other metal ions were determined by fluorescence measurements. A series of metal ion interferences (1.0 × 10^3^ μM) and their mixture were added to a solution of P1 (10 μM). Figure 6 indicated that only the addition of Zn^2+^ showed distinct ratiometric responses. Physiologically important metal ions found in living cells, such as Ca^2+^, Mg^2+^, Na^+^, Fe^2+^ and K^+^, were non-responsive to the probe. Most heavy and transition metal ions, such as Pb^2+^, Hg^2+^, and Cu^2+^, also showed no interference. More importantly, the addition of Cd^2+^, which is recognized as a typical competing ion of Zn^2+^ sensors [2,42,43], led to no obvious variation in the ratio of emission intensity. These results unambiguously demonstrate that the sensing of Zn^2+^ by P1 is hardly affected by common coexisting metal ions even when the concentration of these ions is 100 times higher than that of Zn^2+^ (see Figure 6 caption).

## 4. Conclusions

In conclusion, we have designed and synthesized a new ratiometric fluorescent chemosensor P1 that shows high selectivity, sensitivity to Zn^2+^, and the absence of interference from other competing cations, especially from Cd^2+^. P1 behaves like a tridentate ligand, in which Zn^2+^ is bound with three nitrogen atoms. Zn^2+^/P1 complexation quenches the fluorescence of porphyrin at 650 nm and induces a new fluorescence peak at 610 nm. Moreover, the chemosensor was pH independent under physiological conditions, indicating that it has valuable potential application in biological systems.

## Supplementary Files

Supplementary File 1 Supplementary Information (PDF, 318 KB) A Sensitive Ratiometric Fluorescent Sensor for Zinc(II) with High Selectivity. Sensors 2013, 13, 3131-3141
