# Supplementary material for: A Sensitive Ratiometric Fluorescent Sensor for Zinc(II) with High Selectivity
Source: Sensors (Basel). 2013 Mar 6;13(3):3131–41. doi: 10.3390/s130303131 (PMC3658736; doi:10.3390/s130303131)
Supplement: Supplementary File 1 — Supplementary Information (PDF, 318 KB) [file sensors-13-03131-s001.pdf]

OPEN ACCESS

sensors

ISSN 1424-8220

www.mdpi.com/journal/sensors

Supplementary Information

A Sensitive Ratiometric Fluorescent Sensor for Zinc(II) with High Selectivity. *Sensors* 2013, *13*, 3131-3141

Yuanyuan Lv *, Mingda Cao, Jiakai Li and Junbo Wang *

School of Medicine, Zhejiang University City College, Hangzhou 310015, Zhejiang, China;
E-Mails: cmd92427@163.com (M.C.); 13819162200@163.com (J.L.)

* Authors to whom correspondence should be addressed; E-Mail: lvyy@zucc.edu.cn (Y.L.); wangjb@zucc.edu.cn (J.W.); Tel.: +86-571-8828-4325; Fax: +86-571-8801-8442.

Contents

Figure S1. ^{1}H NMR spectrum of ATPP in $CDCl_3$.

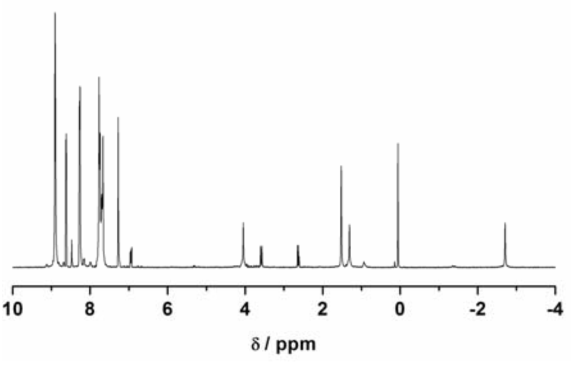

Figure S2. Mass spectrum of ATPP.

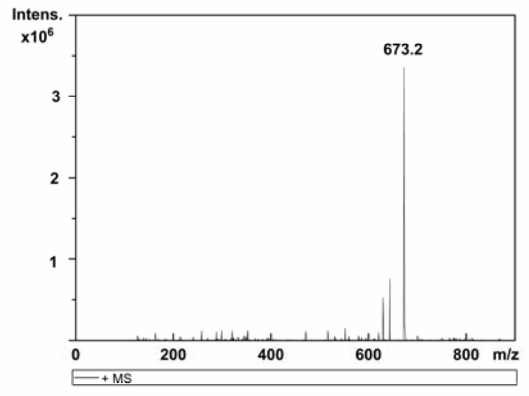

Figure S3. IR (in KBr) spectrum of ATPP.

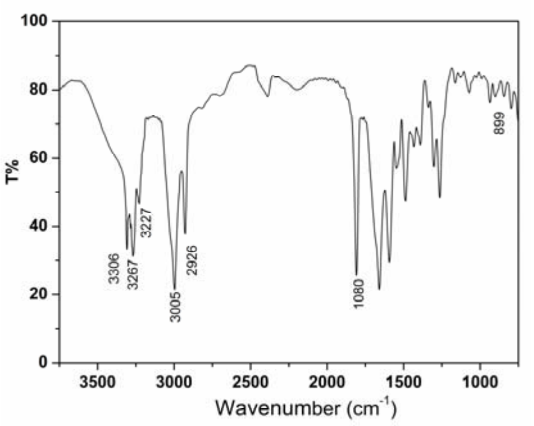

Figure S4. ^{1}H NMR spectrum of P1 in $CDCl_3$.

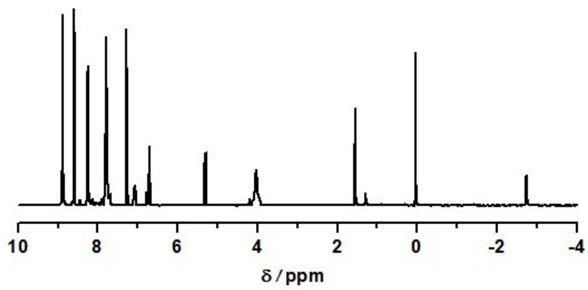

Figure S5. Mass spectrum of P1.

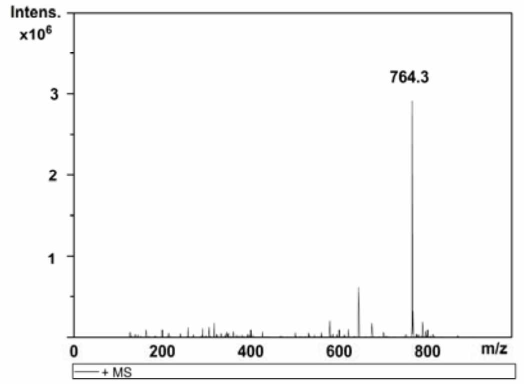

Figure S6. IR (in KBr) spectrum of P1.

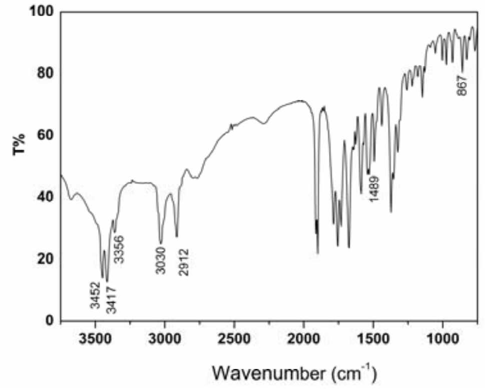

Figure S7. ^{1}H NMR spectra of P1 in the absence (**a**) and presence (**b**) of 1.0 equiv. of Zn^{2+} in DMSO-d_6.

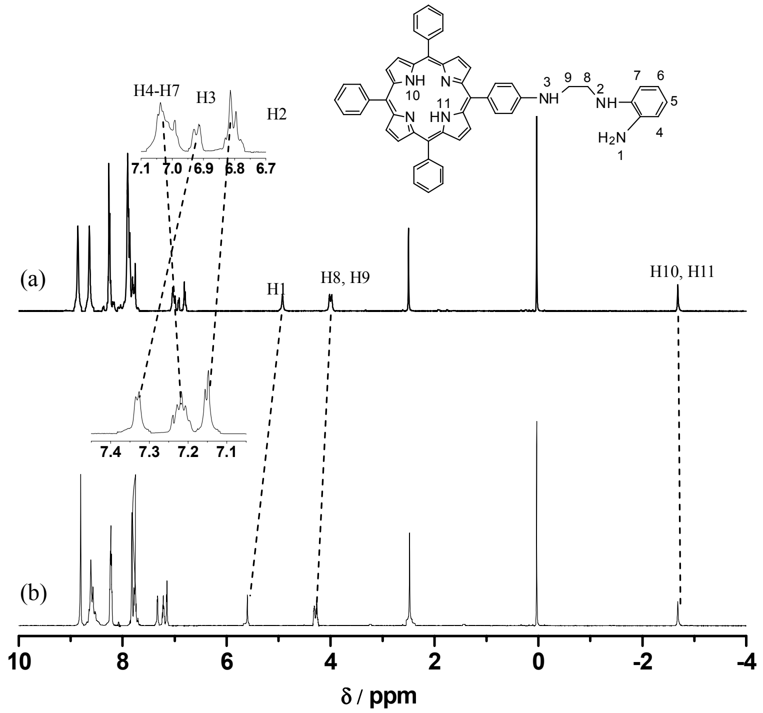

Figure S8. Time evolution of the response of P1 (10 μM) to 2.0 equiv. of Zn^{2+} in EtOH/H_2O solution (1:1, v/v).

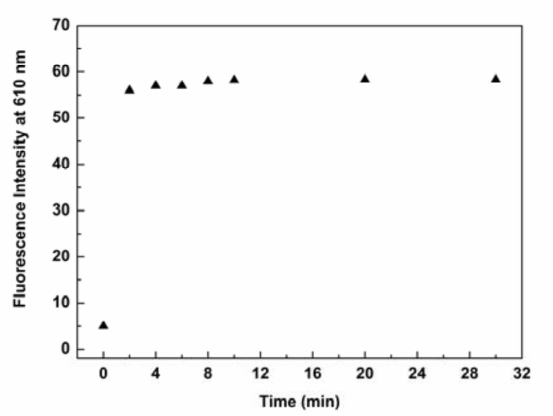

© 2013 by the authors; licensee MDPI, Basel, Switzerland. This article is an open access article distributed under the terms and conditions of the Creative Commons Attribution license (http://creativecommons.org/licenses/by/3.0/).
